# 3D cone-beam CT guidance, a novel technique in renal biopsy—results in 41 patients with suspected renal masses

**DOI:** 10.1007/s00330-012-2498-y

**Published:** 2012-06-02

**Authors:** Sicco J. Braak, Harm H. E. van Melick, Mircea G. Onaca, Johannes P. M. van Heesewijk, Marco J. L. van Strijen

**Affiliations:** 1 Department of Radiology, St Antonius Hospital, PO Box 2500, 3430 EM Nieuwegein, The Netherlands; 2 Department of Urology, St Antonius Hospital, Nieuwegein, The Netherlands

**Keywords:** Percutaneous biopsy, Cone-beam CT, Small renal masses, Outcome, Hard-to-reach

## Abstract

**Objective:**

To determine whether 3D cone-beam computed tomography (CBCT) guidance allows safe and accurate biopsy of suspected small renal masses (SRM), especially in hard-to-reach anatomical locations.

**Materials and methods:**

CBCT guidance was used to perform 41 stereotactic biopsy procedures of lesions that were inaccessible for ultrasound guidance or CT guidance. In CBCT guidance, a 3D-volume data set is acquired by rotating a C-arm flat-panel detector angiosystem around the patient. In the data set, a needle trajectory is determined and, after co-registration, a fusion image is created from fluoroscopy and a slice from the data set, enabling the needle to be positioned in real time.

**Results:**

Of the 41 lesions, 22 were malignant, 17 were benign, and 2 were nondiagnostic. The two nondiagnostic lesions proved to be renal cell carcinoma. There was no growth during follow-up imaging of the benign lesions (mean 29 months). This resulted in a sensitivity, specificity, PPV, NPV, and accuracy of 91.7, 100, 100, 89.5, and 95.1%, respectively. Mean dose-area product value was 44.0 Gy·cm^2^ (range 16.5–126.5). There was one minor bleeding complication.

**Conclusion:**

With CBCT guidance, safe and accurate biopsy of a suspected SRM is feasible, especially in hard-to-reach locations of the kidney.

***Key Points*:**

• *Cone-beam computed tomography has potential advantages over conventional CT for interventional procedures.*

• *CBCT guidance incorporates 3D CBCT data, fluoroscopy, and guidance software*.

• *In hard-to-reach renal masses, CBCT guidance offers an alternative biopsy method.*

• *CBCT guidance offers good outcome and safety and has potential clinical significance.*

## Introduction

The classic but nonspecific symptoms of a renal tumor are loin pain, hematuria, or a palpable mass. In this setting most masses are malignant [1]. This classic triad is rare nowadays. Due to an increase in the use of high-quality abdominal imaging for other, unrelated reasons there is an increasing number of incidentally found renal masses. Many of these masses are asymptomatic and small (<4 cm) renal masses (SRMs). These SRMs tend to behave less aggressively [2, 3]. Thompson et al. [4] found a relationship between tumor size and malignancy. Larger masses have a significantly higher ratio of malignancy. Marinez-Pineiro et al. [5] pointed out the important role in the diagnostic and therapeutic algorithm of biopsying small renal masses for histopathological proof [4] because a substantial percentage of SRMs are benign (13.0–46.3%). Some SRMs can be diagnosed by imaging (e.g., fat in the lesion on CT indicating an angiomyolipoma), but many SRMs cannot be diagnosed based on the imaging alone. This will result in increased indications for performing biopsies.

A new real-time stereotactic needle guidance technique, 3D cone-beam computed tomography (CBCT) guidance, uses a combination of cone-beam CT and real-time fluoroscopy in the angiography suite [6, 7]. Lesions that cannot be clearly identified on ultrasound (US) can be rendered visible. Compared with conventional computed tomography (CT)-guided biopsy, CBCT guidance offers more sterile workspace and better angulation/rotation ability because of the C-arm configuration, making it easier to biopsy hard-to-reach SRMs, especially in the upper pole and/or on the anterior side of the kidney [7]. The objective of this study is to determine the outcome of biopsying SRMs, especially hard-to-reach lesions, in a prospective cohort of patients.

## Materials and methods

This study was approved by the institutional review board. Informed consent was obtained from all patients. Between October 2006 and November 2009 we performed 43 procedures in 43 patients using CBCT guidance. There were 28 men and 15 women, with a mean age of 61.9 years (range 35–81). The baseline data of the study population are shown in Table 1. These patients had undergone abdominal imaging for nonurogenital issues, during which a suspicious renal mass was discovered as an incidental finding. Based on the abdominal imaging, the lesions were not (easily) accessible under CT guidance because of the anatomical position of the lesion. Before referring these patients for CBCT guidance, the patients were evaluated by ultrasound. Only patients with lesions inaccessible under ultrasound guidance (due to body habitus, mass location, invisibility due to gas or bone, or interposition of other organs) were included in this study. There was no target lesion size limit. During CBCT guidance, patients had to be able to lie reasonably still for a short period of time (approximately 30 min) and comply with breath-hold commands if needed. Furthermore, patients with contraindications for percutaneous intervention (e.g., blood coagulation disorders) were excluded.

**Table 1:** The baseline data of the study population

Characteristic	Value
Age (years), mean ± SD	62.0 ± 11.3
Male/female (*n*)	26/15
Body mass index (kg/m^2^), mean ± SD	27.1 ± 3.5
Diameter of lesion (mm), mean ± SD	25.0 ± 7.6
Number of biopsies per procedure, mean ± SD	3.4 ± 0.7
Lesion solid/cystic/both (*n*)	38/1/2
Procedure time (min), mean ± SD	29.2 ± 10.7
Dose area product (Gy·cm^2^), mean ± SD	44.0 ± 21.0

### Procedure

**Fig. 1a, b:**
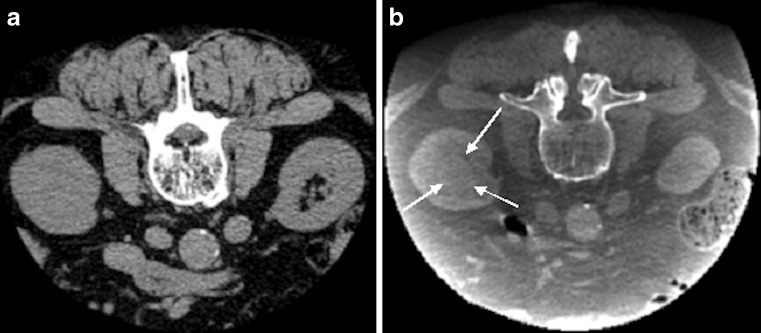
An 81-year-old man with a suspected endophytic kidney mass during abdominal CT imaging. After biopsy, histopathological analysis revealed a clear cell renal cell carcinoma. Difference in cone-beam CT (CBCT) without contrast medium (**a**) and contrast-enhanced CBCT (**b**). The endophytic renal mass (*white arrows*) is only visible on the contrast-enhanced CBCT

**Fig. 2a–d:**
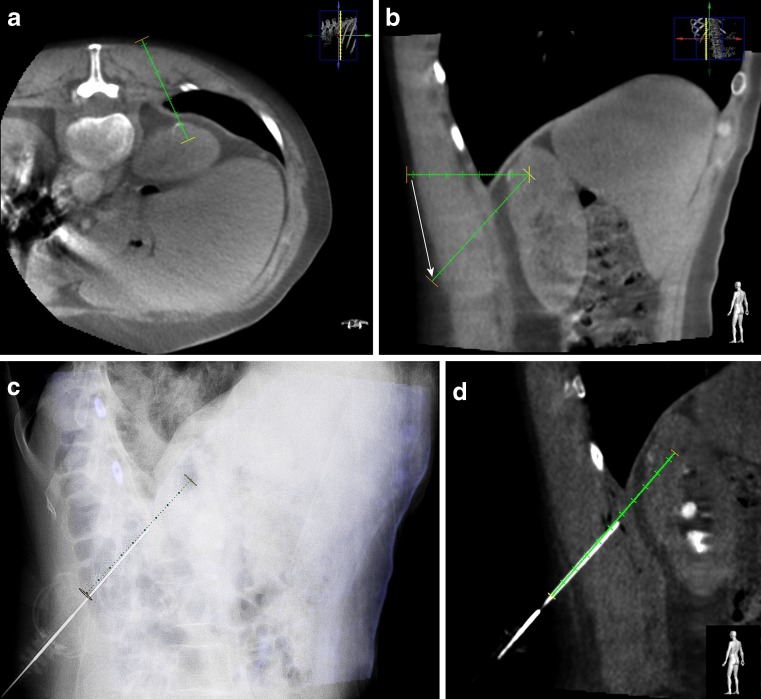
A 73-year-old man with a suspected lesion at the dorsal side of the upper pole of the left kidney. Histopathological analysis revealed a clear cell renal cell carcinoma. Cone-beam computed-tomography (CBCT) guidance sequences during a procedure. **a** After obtaining a CBCT the radiologist determines in the axial CBCT the desired needle trajectory in the axial view (avoiding the costae). **b** The needle trajectory is adjusted by the radiologist (*white arrow*) to avoid essential organs (the diaphragm/lung). **c** Fusion image of fluoroscopy and the double oblique cone-beam CT slice with the predefined needle trajectory, making real-time accurate needle placement possible. **d** Control cone-beam CT image (double oblique) with the guiding cannula in place over the predefined needle trajectory, checking needle position and possible complications

CBCT guidance uses a flat panel detector C-arm system (XperCT® and XperGuide®, Allura FD20, Philips Healthcare, the Netherlands). A 3D volume data set is acquired during a 240° rotation of the C-arm around the upper abdomen of the patient in 4–10 s. In six patients, intravenous contrast (50 mL with 4 mL/s Xenetix® 300 mg/mL, Guerbet, the Netherlands; delay before data acquisition of 40 s) was administered during the examination to discriminate the mass from surrounding structures/parenchyma (Fig. 1). For all other masses, no contrast administration was needed because they were exophytic or had other discriminating factors. The radiologist determines a safe needle trajectory within the reconstructed data set, taking account of critical anatomical structures (Fig. 2a, b). Using the information of the planned needle trajectory, a fusion image of fluoroscopy and the relevant double oblique slice of the cone-beam CT is created in which the needle can be accurately positioned. The patient is asked to breathe in until the diaphragm is in the same position as the double oblique slice of the CBCT. When the diaphragm on the fluoroscopy image matches the CBCT slice during inspiration, the patient was given a breath-hold command, and under real-time fluoroscopy the needle is advanced over the predefined needle path to the right depth (Fig. 2c) [6, 7]. The procedure is performed with local anaesthesia. Sampling was done using a coaxial technique with a guiding cannula [Bard® TruGuide®;17 G; 13 or 17 cm (Bard Biopsy Systems, Tempe, AZ, USA)] positioned just proximal to the renal mass. Three to six 18 G biopsies were then taken through the guiding needle to obtain at least 1 cm of biopsy length. The Tru-Cut needle (Bard® Magnum®;18 G; 16, 20 or 25 cm; side cutting, 15 or 22 mm) is loaded in an automated biopsy gun (Bard®).

After the needle intervention, a (collimated) control cone-beam CT was always performed for checking needle position accuracy and to check for possible complications (Fig. 2d). The procedures were performed by an interventional radiologist (M.S., >10 years of experience) and a radiology resident (S.B., 5 years of experience), both with equal experience in CBCT guidance. Local experience in using this CBCT technique is now 3 years. After the procedure, patients remained under observation in the hospital for 4 h. In case of significant changes in vital signs or clinical status, repeat abdominal imaging was indicated (e.g., ultrasound or CT depending on the clinical condition of the patient).

### Data collection and analysis

All the radiological reports, histopathological data, and medical records were collected for each procedure. The result of a percutaneous renal mass biopsy was defined as true positive (TP) if the histopathological examination revealed a renal cell carcinoma or a metastasis. A false positive (FP) biopsy was defined as a biopsy showing malignancy, where there was no evidence of a malignancy during surgery [in the absence of preoperative chemotherapy of other (ablative) therapy]. If the histopathology showed a benign result (e.g., normal renal parenchyma, infection, infarction, or oncocytoma), the biopsy was considered true negative (TN) if the renal mass proved to be benign during surgical resection or if there was no suspicion of a malignancy during follow-up imaging for at least 12 months [8]. If the final diagnosis during surgery showed malignancy and the diagnosis based on the results of the biopsy procedure was benign or nondiagnostic, the biopsy result was defined as false negative (FN). Sensitivity, specificity, positive predictive value (PPV), negative predictive value (NPV), and diagnostic accuracy were calculated. The dose-area product (DAP) (Gy·cm^2^), which was obtained from the system, was also registered for an indication of the radiation dose involved. Data registration was performed using Excel (Microsoft® Office Excel, Redmond, WA, USA). The outcome was determined by using a 2 × 2 table.

## Results

The sensitivity, specificity, PPV, NPV, and accuracy of CBCT guidance in renal masses were 91.7, 100, 100, 89.5, and 95.1%, respectively (Table 2). In 41 biopsies, 22 (53.7%) malignancies were found: 10 clear cell renal cell carcinomas (RCCs), two papillary RCCs, one chromophobe RCC, one eosinophilic RCC, one sarcomatoid type RCC, four transitional cell carcinomas, two metastases of non-small-cell lung cancer, and one B-cell lymphoma. All of these except the metastases and lymphoma were also surgically proven. During follow-up imaging of these patients, no evidence of tumor seeding on the needle track was seen. Seventeen (41.5%) biopsies, without rebiopsying, were classified as benign: four post-infectious changes, three oncocytomas, six biopsies showing normal renal parenchyma, one organizing hematoma, one angiomyolipoma, one inflammatory pseudotumor, and one infarction. The mean follow-up of the benign lesions was 29 months (range 18–45), without evidence of malignancy (e.g., lesion size growth). Two (4.9%) were nondiagnostic biopsies.

**Table 2:** Outcome of percutaneous biopsy of small renal masses using 3D cone-beam computed tomography (CBCT) guidance

CBCT guidance	Renal mass		Total
	Malignant	Benign	
Malignant	22	0	22
Benign	2	17	19
Total	24	17	41

The two patients with nondiagnostic lesions of 23.3 and 31 mm underwent (partial) nephrectomy because of suspicious cells found during histopathological examination. These masses proved to be RCC after surgical resection.

Twenty-two (53.7%) lesions were endophytic. Contrast was used to visualize the lesion on CBCT in six patients (14.6%). Detailed information on tumor location is shown in Table 3. Mean diameter was 25.0 mm (range 10–40 mm), and the mean number of biopsies per procedure was 3.4 (range 3–6). Mean DAP value was 44.0 Gy·cm^2^ [standard deviation (SD) 21.0; range 16.5–126.5]. There were no serious adverse events. In one patient (2.4%) a continuing minor bleeding through the co-axial needle was present. This was treated directly by injecting hemostatic material (Spongostan®; Baxter, Deerfield IL, USA)]. No additional intervention or prolonged hospital admission was required.

**Table 3:** Anatomical details of tumor location

	Lower half	Upper half	Total
Dorsal	2	11	13
Lateral	–	8	8
Medial	–	5	5
Ventral	7	8	15
Total	9	32	41

## Discussion

CBCT guidance has a sensitivity of 91.7%, an NPV of 89.5%, and an accuracy of 95.1% for histopathological biopsies of renal masses. To the authors’ knowledge, only Kroeze et al. [9] has so far described CBCT guidance for biopsy of renal masses. They reported a technical feasibility of 77% in a small patient population (*n* = 13) [9]. We report a better outcome, probably because of our larger population and longer experience with CBCT guidance.

Volpe et al. [10] reviewed the technique, safety, and accuracy of sampling of renal tumors by core biopsy using CT or US and reported a sensitivity of 70–100%. In the study performed by Rybicki et al. [11], a sensitivity of 90% was reported. Several other studies have also evaluated the sensitivity of renal biopsies, resulting in an overall sensitivity for diagnosis of malignancy of 80–95%. However the studies show considerable variation in population and method of guidance (US or CT) [1, 12–14].

Vasudevan et al. [1] report 47 malignant biopsies, 23 benign biopsies, and 4 false negative biopsies resulting in an NPV of 85.2%. Rybicki et al. [11] had an NPV of 64% in their population. In our study population, the NPV was 89.5%.

Nadal et al. [12] described an accuracy of 87% on the initial biopsy improving to 97% after a second biopsy. Biopsies were performed using an 18 G core biopsy needle, and an overall accuracy of 89% was achieved [15, 16]. Shannon et al. [17] reported a diagnostic biopsy rate of 78%, with 22% nondiagnostic biopsies due to insufficient material or nonmalignant renal material. Our accuracy is in the same range (95.1%).

Our results of the percutaneous renal biopsies using CBCT guidance are comparable with those in the literature. However in most of our procedures the needle trajectory had to be at a (steep) double oblique angle, which is more difficult to perform using CT(fluoroscopy) guidance. To perform angulated procedures with CT guidance, there are a couple of techniques available. One possibility is the gantry tilting method, whereby the gantry of the CT system is tilted between 0 and 30° depending on the vendor, making an angulated biopsy possible. The tilting method proved to be accurate (90–96%) in biopsy of hard-to-reach upper abdominal masses [18]. The operator can, during the tilting method, also use CT fluoroscopy, visualizing the slices of interest in real time. A drawback of tilting is the negative influence on the sterile workspace, which is already reduced during conventional CT (fluoroscopy) guidance compared to the C-arm configuration [19].

Another technique, which has a histopathological accuracy of 76%, is the triangulation technique as described by vanSonnenberg et al. [20]. It requires calculation of the angle and trigonometric tables. During the procedure a large number of slices need to be scanned to visualise the whole needle trajectory, resulting in longer procedure times and more radiation exposure to the patient. Because of the long needle trajectory, the needle passes different soft tissues with different resistance; this, in combination with respiratory movement, adds more complexity to the procedure [21].

For angulated procedures, MRI also offers a good alternative to a needle intervention, especially for lesions invisible on CT and US. Stattaus et al. [22] reported a sensitivity of 95.5%, specificity of 100%, and an accuracy of 96% using a short, wide-bore 1.5 T MR system. Kühn et al. [23] reported an accuracy of 94%. Both groups agree that MR-guided procedures are also feasible for lesions with quite a steep angulation due to MRI’s capacity for multiplanar viewing. However, they acknowledge that the gantry size in regular MR systems is limited. Other possible disadvantages of MR-guided procedures include that they are considered expensive because of dedicated materials and biopsy systems, the reported mean procedure time is 42–48 min (compared to our 29 min), and needle artifacts may be present.

In our experience steep, double-angulation procedures are easier with CBCT guidance because of the C-arm geometry with an angulation range up to 50°. In addition to this, there is real-time feedback of the fluoroscopy with a large field-of-view compared to CT fluoroscopy. Breathing can be halted at a point where the diaphragm on the fluoroscopy image exactly matches the diaphragm in the (double oblique) overlay slice of the cone-beam CT. This enables real-time compensation of breathing movements during the progression of the needle, compensating for the respiratory movement and deviation due to different tissue resistance. Therefore, in our experience, CBCT guidance is better for biopsying hard-to-reach lesions than CT or MRI.

The two nondiagnostic lesions presented normal kidney parenchyma with atypical cells, but the biopsies were found to be insufficient for diagnosis in the histopathological report. Because of this report and the malignant features on abdominal imaging with CT, the decision was made to perform a (partial) nephrectomy. Both of these lesions were exophytic. The definite diagnosis in both patients was a renal cell carcinoma. Checking with the control CBCT, it appears that the biopsies were most likely planned slightly peripheral to the lesions. That is a possible reason for the presence of at least some atypical cells in the specimen suggesting a malignancy, but there was not enough material to make a definitive conclusion.

The comparable results of CBCT guidance and the literature suggest that this new technique can be performed easily and accurately, but it is not as widely available as CT, ultrasound, or MRI.

Using the reported conversion factor from DAP to effective dose of 0.28–0.29 (mSv·Gy^−1^·cm^−2^) by Suzuki et al. [24] during abdominal cone-beam CT, our mean effective dose was 12.5 (± 5.9 SD) mSv. This conversion was done to compare the dose of CBCT guidance to CT guidance in the literature. Tsalafoutas et al. [25] report an effective dose of 23 mSv during conventional CT-guided biopsies in which unenhanced, intervention, and control post-procedure CT data acquisitions were performed. Other studies report a lower effective dose (7.1–12 mSv) during CT-guided biopsies; however in these procedures no final post-procedure CT data acquisition was performed to check for possible complications [26, 27].

No serious complications occurred during our procedures. The literature on renal mass biopsy shows complications of pain, hematuria, bleeding, and tumor seeding. The percentage of complications depends on the size of the needle [28, 29]. Vaseduvan et al. [1] used a 16 G needle and reported a complication rate in 100 biopsies of 1%, for which the patient needed a blood transfusion. Nadal et al. [12] reported a complication rate of 12% in biopsy of renal masses using an 18 G biopsy needle. In this report 3% of the procedures needed a blood transfusion. In the report of Whittier et al. [30], a 13% overall complication rate was reported using a 14 G biopsy needle. Fifty percent of these were major complications (e.g., gross hematuria, death). In our population we had one patient (2.4%) with some persistent bleeding out of the co-axial needle (17 G), which could be directly treated. No other complications or tumor seeding along the needle track was noticed during the follow-up.

A limitation of this study is the inability to obtain a definitive confirmation of the lesions defined as nonmalignant (except the two non-diagnostic lesions with suspicious cells which were operated), e.g., organising hematoma, pseudotumor, and the three nondiagnostic results, because all were treated conservatively. However, no changes on subsequent CT indicating malignancy (e.g., increasing size) were noted during follow-up, and therefore the definitive diagnosis was assumed to be benign. A second possible limitation is our relatively short follow-up period of 2 years.

A new image-merging feature has recently been introduced into the planning system (XperGuide®, Philips Healthcare, the Netherlands). This tool allows recent cross-sectional DICOM data (CT or MRI) to be used to plan the needle trajectory. After importing these data into the system, a match is made manually between the recent DICOM data and the (low-dose) cone-beam CT data. Preliminary experimental results of this merging feature for accurate planning are promising, possibly leading to even higher accuracy, especially in endophytic masses.

In conclusion, our results demonstrate acceptable sensitivity and accuracy of renal mass needle biopsy using 3D CBCT guidance. CBCT guidance appears to be a safe and accurate procedure for biopsy of small (<4 cm) renal masses, especially those in difficult-to-reach anatomical regions.
